# PReS-FINAL-2165: Pharmacogenetic determinants of response to methotrexate in juvenile idiopathic arthritis

**DOI:** 10.1186/1546-0096-11-S2-P177

**Published:** 2013-12-05

**Authors:** S Pastore, V Moressa, G Stocco, G Decorti, L Lepore

**Affiliations:** 1 University of Trieste, Trieste, Italy; 2 Department of Life Sciences, University of Trieste, Trieste, Italy; 3 Institute for Maternal and Child Health - IRCCS "Burlo Garofolo", Trieste, Italy

## Introduction

Juvenile idiopathic arthritis (JIA) is the most common arthritis disease of childhood and is an important cause of disability. Methotrexate (MTX) is the mainstay treatment in JIA. Unfortunately, 30-35% of patients fail to respond MTX, and the delay in identifying the optimal treatment at an early stage of disease can lead to long-term joint damage. Recent studies have evaluated comprehensively the effect of genetic variants in candidate genes involved in MTX pharmacokinetics and pharmacodynamics on the response to the medication in children with JIA. These studies seem to indicate that the most relevant variants to predict MTX response in JIA are those in ATIC, ITPA and SLC19A1 genes.

## Objectives

To evaluate the role of these candidate genetic factors on the attainment of clinical remission on MTX in an Italian cohort of children with JIA.

## Methods

Patients with JIA treated with MTX were enrolled by the Pediatric Clinic of Burlo Garofolo Children's Hospital in Trieste; clinical data was collected retrospectively from patients' charts. Clinical remission on MTX for 6-month-period was evaluated according to Wallace criteria. The most relevant functional SNP for each gene considered was characterized by Taqman (rs2372536 in ATIC, rs1127354 in ITPA) or PCR-RFLP (rs1051266 in SLC19A1) assays on patients' DNA extracted from peripheral blood.

## Results

Complete analysis was performed on 69 patients. Of these, 76.8% were female, median age at MTX start was 8 years (range 1 - 22). JIA presentation was oligoarticular in 63.7%, poliarticular in 33.3% and enthesitic/psoriatic in 2%. At the beginning of MTX therapy, disease had been lasting for a median of 1 year (range 0 - 19); MTX was administered at a median dose of 15 mg/m2 (range 10 - 20), subcutaneously in 62.3% of patients and orally in the rest. Genotyping showed minor allele frequencies of 36.2% for rs2372536, 5.1% for rs1127354 and 49.3% for rs1051266, consistent with previous reports in Europeans. Assessment of response to MTX showed that 27.5% of reached remission stable for 6 months. No statistically significant effect of the demographic and clinical covariates was found on MTX response. However, genotyping analysis identified a significant association between the GG variant of ATIC rs2372536 and improved response to therapy: frequency of this genotype was 32% among patients with stable remission and 6% among those with no stable remission (p = 0.010). For the variant of ITPA rs1127354, an effect was present in the opposite direction: the A allele was present present in none of the patients with stable remission and in 12% of those without stable remission (p = 0.04). Preliminary analysis of SLC19A1 rs1051266 revealed a trend for an association of the variant with increased response (p = 0.06). Multivariate analysis supports the independent effect of the genotypes considered on methotrexate response.

## Conclusion

This report supports the utility of genotyping candidate genes to predict MTX response in children with JIA and should be further validated clinically by larger and prospective studies.

## Disclosure of interest

None declared.

